# A spectroscopy based prototype for the noninvasive detection of diabetes from human saliva using nanohybrids acting as nanozyme

**DOI:** 10.1038/s41598-023-44011-y

**Published:** 2023-10-12

**Authors:** Lopamudra Roy, Susmita Mondal, Neha Bhattacharyya, Ria Ghosh, Amrita Banerjee, Soumendra Singh, Arpita Chattopadhyay, Saleh A. Ahmed, Rabab S. Jassas, Munirah M. Al-Rooqi, Ziad Moussa, Ismail I. Althagafi, Debasish Bhattacharya, Kallol Bhattacharya, Asim Kumar Mallick, Samir Kumar Pal

**Affiliations:** 1 Department of Applied Optics and Photonics, University of Calcutta, JD-2, Sector-III, Salt Lake, Kolkata, West Bengal 700 106 India; 2 Department of Chemical and Biological Sciences, S. N. Bose National Centre for Basic Sciences, Kolkata, 700106 India; 3 Department of Radio Physics and Electronics, University of Calcutta, Kolkata, 700009 India; 4 Department of Physics, Jadavpur University, 188, Raja Subodh Chandra Mallick Rd, Poddar Nagar, Jadavpur, Kolkata, West Bengal 700032 India; 5 Neo Care Inc, 27, Parker St, Dartmouth, NS B2Y 2W1 Canada; 6 Electrical and Computer Engineering Department, Dalhousie University, 6299 South St, Halifax, NS B3H 4R2 Canada; 7 Department of Basic Science and Humanities, Techno International New Town Block, DG 1/1, Action Area 1 New Town, Rajarhat, Kolkata, 700156 India; 8 Department of Physics, Sister Nivedita University, DG 1/2 New Town, Action Area 1, Kolkata, 700156 India; 9 Chemistry Department, Faculty of Applied Science, Umm Al-Qura University, 21955 Makkah, Saudi Arabia; 10 Chemistry Department, Faculty of Science, Assiut University, Assiut, 71516 Egypt; 11 Department of Chemistry, Jamoum University College, Umm Al-Qura University, 21955 Makkah, Saudi Arabia; 12 Department of Chemistry, College of Science, United Arab Emirates University, P.O. Box 15551, Al Ain, Abu Dhabi United Arab Emirates; 13 Department of Gynecology & Obstetrics, Nil Ratan Sircar Medical College & Hospital, 138, AJC Bose Road, Sealdah, Raja Bazar, Kolkata, 700014 India; 14 Department of Pediatrics, Nil Ratan Sircar Medical College and Hospital, Kolkata, 700014 India

**Keywords:** Biomarkers, Diseases, Health care, Engineering, Nanoscience and technology, Optics and photonics

## Abstract

The recent prediction of diabetes to be a global pandemic invites a detection strategy preferably non-invasive, and bloodless to manage the disease and the associated complications. Here, we have synthesized chitosan polymer functionalized, organic–inorganic bio-compatible nano-hybrids of Mn_3_O_4_ nanoparticles, and characterized it by utilizing several optical methodologies for the structural characterization which shows the Michaelis Menten (MM) kinetics for glucose and alpha-amylase protein (well-known diabetes biomarkers). We have also studied the potentiality for the detection of alpha-amylase in human salivary secretion which is reported to be strongly correlated with uncontrolled hyperglycemia. Finally, we have developed a prototype for the measurement of glucose (LOD of 0.38 mg/dL, LOQ of 1.15 mg/dL) and HbA1c (LOD of 0.15% and LOQ of 0.45%) utilizing the basic knowledge in the study for the detection of uncontrolled hyperglycemia at the point-of-care. With the limited number of clinical trials, we have explored the potential of our work in combating the diabetic pandemic across the globe in near future.

## Introduction

Diabetes is a chronic human disorder due to an imbalance of blood sugar affecting more than 422 million people worldwide^1^. While diabetes can cause several comorbidities including neuropathies, and blindness, it involves directly or indirectly 1.6 million deaths every year according to World Health Organization (WHO)^2^. Thus, frequent measurement of blood sugar is crucial for proper glycemic management. Normally, the management of diabetes is associated with the direct measurement of blood glucose several times a day^3,4^. Although direct monitoring of blood glucose is the most accurate technique, it suffers from mental trauma for patients due to pain as well as infection/inflammation of the fingertip. Thus, the demand for the technology for non-invasive detection of blood glucose is the need of the hour. From the literature it is evident that Nano-technology has played a significant role in the development of Nano-material based non-invasive blood glucose monitoring methods in the last 15 years as summarized in Table 1. In addition, Nano-material based diabetes sensors have expanded from blood test to utilizing bodily fluids including sweat, tears, urine, saliva and intestinal fluids^1^.

**Table 1:** Literature study on non-invasive blood glucose monitoring based on Nano-technology.

Element/method of detection	Nanomaterial used	Detection medium	Limit of detection	References
Peroxidase mimetics for colorimetric detection	ZnFe_2_O_4_ magnetic nanoparticle	Human urine	3.0 × 10^−7^ mol L^−1^	^5^
γ-Fe_2_O_3_ /TMB/glucose–glucose oxidase	γ-Fe_2_O_3_ nanoparticles	Human blood and urine	0.21 μM	^6^
Enzyme-responsive plasmonic bimetallic nanoshell functionalized with glucose oxidase/UV–vis absorption	Hollow Ag/Au bimetallic nanoshell	Serum	Not available	^7^
Glucose oxidase (GOx)-mediated oxidative etching of gold nanorods/colorimetric assay	Gold nanorods	Serum	0.1 mM	^8^
Glucose oxidase mediated etching of Au–Ag NPs/UV–Vis absorption	Au–Ag nanoparticles	Blood and urinary glucose	Not available	^9^
Glucose oxidase-functionalized etching of gold nanoclusters conjugate/Fluorescence spectra	Tetrakis (hydroxylmethyl) phosphonium-protected gold nanoparticlecluster	Serum and urine sample	0.7 × 10^−6^ M	^10^
Glucose/Carbon-dot based fluorescent glucose sensing motif	Boronic acid modified carbon dots	Human serum	1.5 μM	^11^
Glucose oxidase- and peroxidase-like dual active MnO_2_ nanoflakes as nanoenzyme Colorimetric detection of glucose using one-pot enzyme-free cascade catalysis	MnO_2_ nanoflakes	Human blood serums	1 × 10^−6^ m	^12^
Peroxidase-mimic Naannozymes/colorimetric based	Ag nanoparticles within the 3D matrix of a cotton fabric	Urine	0.08 mM	^13^
Glucose oxidase-conjugated graphene oxide/ MnO_2_ nanozymes/colorimetric assay based	Graphene oxide/MnO_2_ nanozymes	Serum/ whole blood	3.1 mg/dL	^14^
Peroxidase Mimic/Colorimetric Detection	Polyethyleneimine-stabilized platinum NPs	Saliva	4.2 μM	^15^
Reshaping approach of plasmonic multibranched gold nanostructures/Colorimetric Detection	Gold nanostructures	Saliva	0.4 mg/dL	^16^

Although, the term nano-enzyme is related to hybrid nano-particle containing organic molecule- functionalized inorganic materials, it has been solidified into an enzyme-mimicking Nano-material that demonstrated intrinsic redox-like activity^17,18^. The nano-enzymes are reported to have several significant advantages including simple and excellent tenable catalytic activity, controllable synthesis protocol, high environmental stability on modification low-cost and scale-up possibility^19,20^. Recently, various types of nanoparticles^21^, wearable biosensors^22–24^, nano-enzymes containing carbon, Nano-tube and graphene oxides^25^, polymer coated nano-particles^26,27^, and nanohybrids^27^ are spotted to have the potential for the bio-detection. Earlier, several attempts have been made to show that glucose in the body fluids correlates with that of the whole blood^19^. As shown in Table 1, various non-invasive studies in diabetes diagnosis using nano-technology have attempted using different body fluids. However, multiple considerations are required to assure the quality and accuracy of glucose concentration in body fluids. One of the main factors is that glucose concentration in these body fluids is lower than that in whole blood i.e. where 2–40 mM in blood, 0.008–1.77 mM in saliva, 0.012–1.11 mM in sweat, 0.05–5 mM in tears, and 1.99–22.2 mM in ISF^19,28^. Therefore, higher sensitivity of the proposed non-invasive and alternative diagnostic technique is the natural demand. Among the other body fluids saliva can be collected easily by spitting but care should be made for the impurities in the samples. From the biochemistry point of view, saliva is a mixture of 99.5% water and 0.5% electrolytes (glycoproteins, lipase, mucin, amylase, glucose, and antimicrobial enzymes)^29^. In this study, we have explored the unique property of a nano-enzyme for the detection of alpha-amylase, a well-known diabetes marker from saliva and well-known for its strong correlation with uncontrolled hyper-glycemia, or higher value of HbA1c in human subjects.

**Figure 1:**
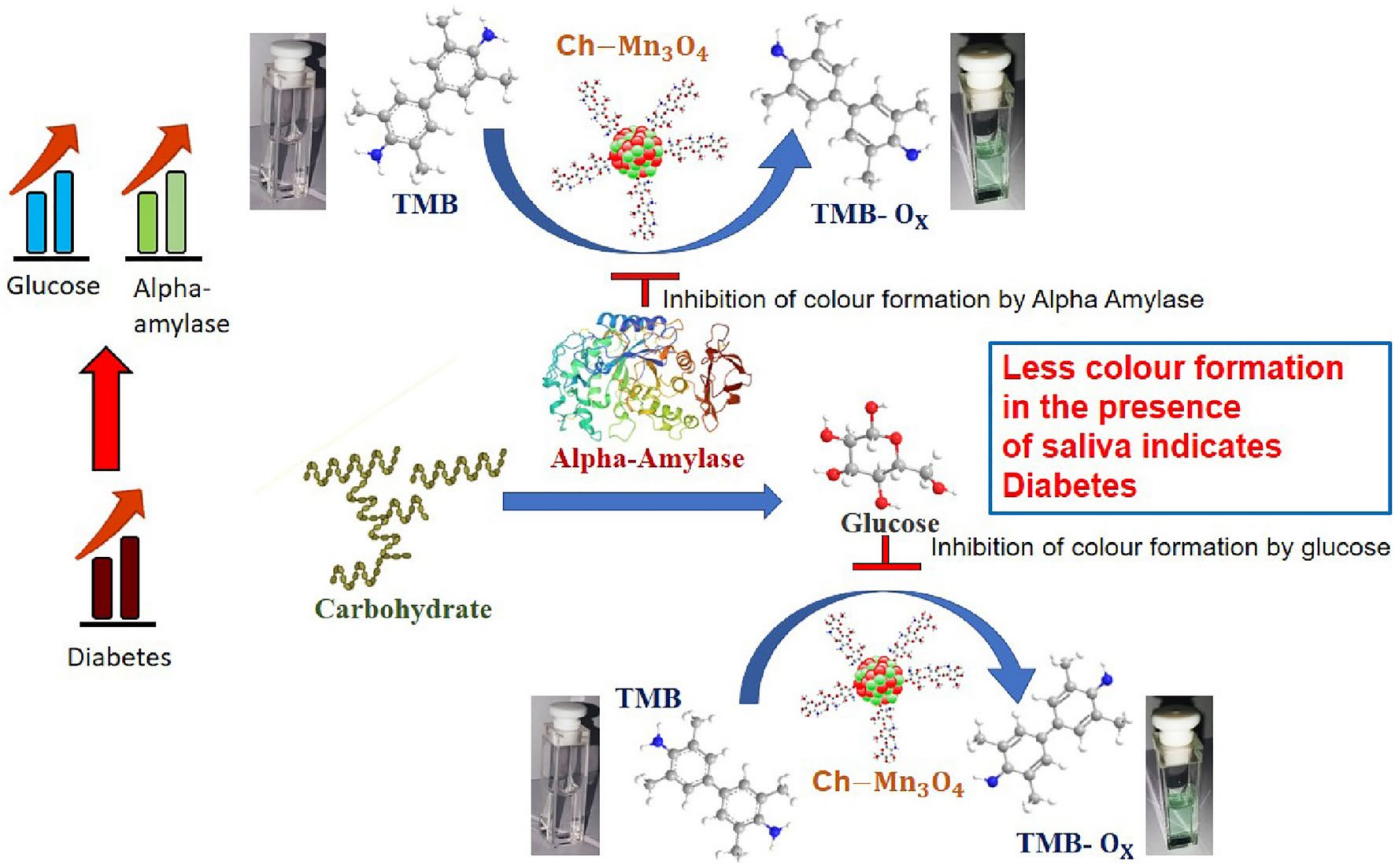
Schematic presentation of the detection mechanisms using the Ch-Mn_3_O_4_ nanozyme.

In our present study, we have synthesized chitosan (Ch) capped Mn_3_O_4_ Nano-enzymes for the detection of blood glucose from human saliva. High-resolution electron microscopy, Fourier Transform Infrared Spectroscopy (FTIR), Raman Scattering, Dynamic Light Scattering (DLS), and Zeta-potential measurements have been used for the characterization of the nano-enzyme. Michaelis–Menten kinetics of the nano-enzyme on colorimetric substrate 3,3′,5,5′-Tetramethylbenzidine (TMB) clearly shows the enzymatic behavior of the Nano-material which depends on the presence of glucose and other enzymes namely, amylase which is present in human saliva in abundance. Amylase is one of the important enzymes for food digestion. Human body gets serum amylase mainly from the salivary gland and pancreatic gland secretions^30^. In diabetes, due to the increased cell resistance to endogenous insulin and/or the defective insulin secretion, less amount of pancreatic amylase is contributed in serum amylase^31,32^. On the other hand, due to the dysfunction of the salivary glands (parotid, submandibular, and sublingual), it shows altered expression of amylase and cyclic adenosine monophosphate (cAMP) which lead to increase the salivary amylase^33–36^. Although, there is a controversy on the salivary amylase level to be low^37,38^, high^39–41^, and comparable^42,43^ for the diabetes patients. However, our study has the positive correlation between the salivary amylase with the diabetes. In Fig. 1, the mechanisms of the present work are schematically depicted. We have also developed a prototype using the nanozyme for the analysis of saliva at the point of care. With our limited clinical trial using the prototype, we attempted to correlate not only blood glucose in human blood with that in saliva but also detect glycated haemoglobin (HbA1c) present in the whole blood from the enzymatic activity of the nanoenzyme in presence of saliva. To our understanding, our studies find a significant relevance in the contemporary nanoenzyme-base colorimetric bio-sensor for non-invasive glucose monitoring.

## Results and discussion

### Characterization of chitosan functionalized Mn_3_O_4_ nanoparticles (Ch-Mn_3_O_4_ NPs)

**Figure 2:**
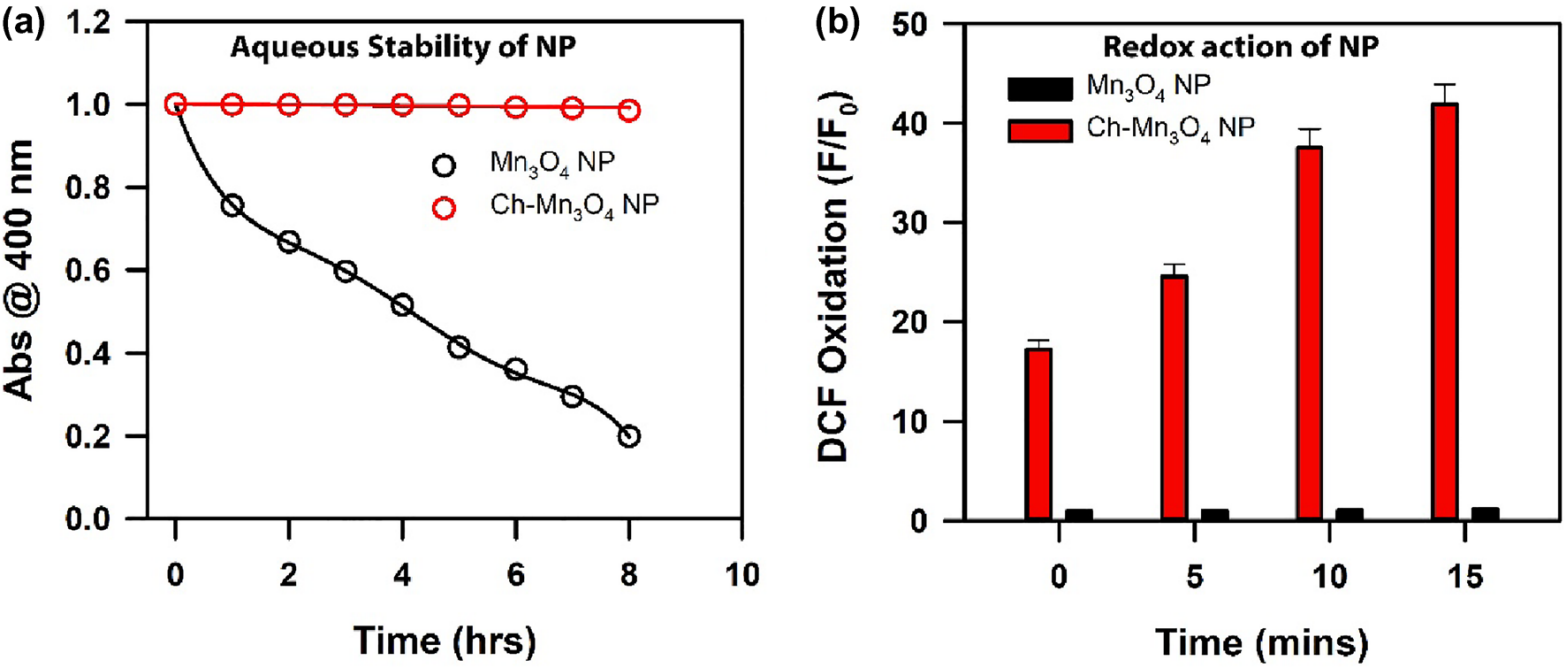
(**a**) Aqueous stability of uncapped Mn_3_O_4_ nanoparticle and chitosan capped Mn_3_O_4_ nanoparticle. (**b**) Redox action of uncapped Mn_3_O_4_ nanoparticle and chitosan capped Mn_3_O_4_ nanoparticle.

**Figure 3:**
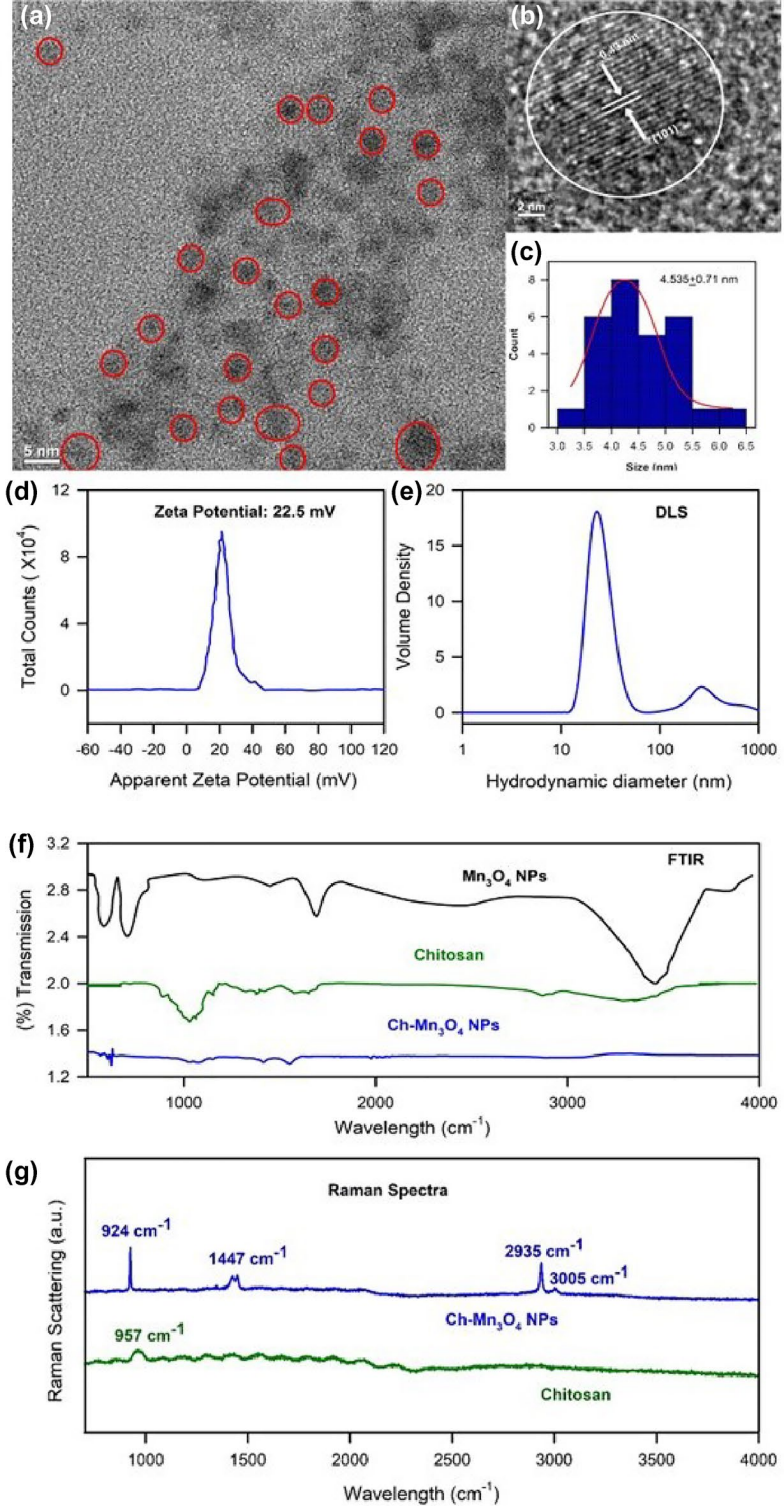
Characterization of Ch-Mn_3_O_4_ nanoparticles (**a**) TEM image, (**b**) HRTEM, (**c**) the size distribution collected from HRTEM, (**d**) zeta potential, (**e**) DLS, (**f**) FTIR, and (**g**) Raman Spectra.

**Figure 4:**
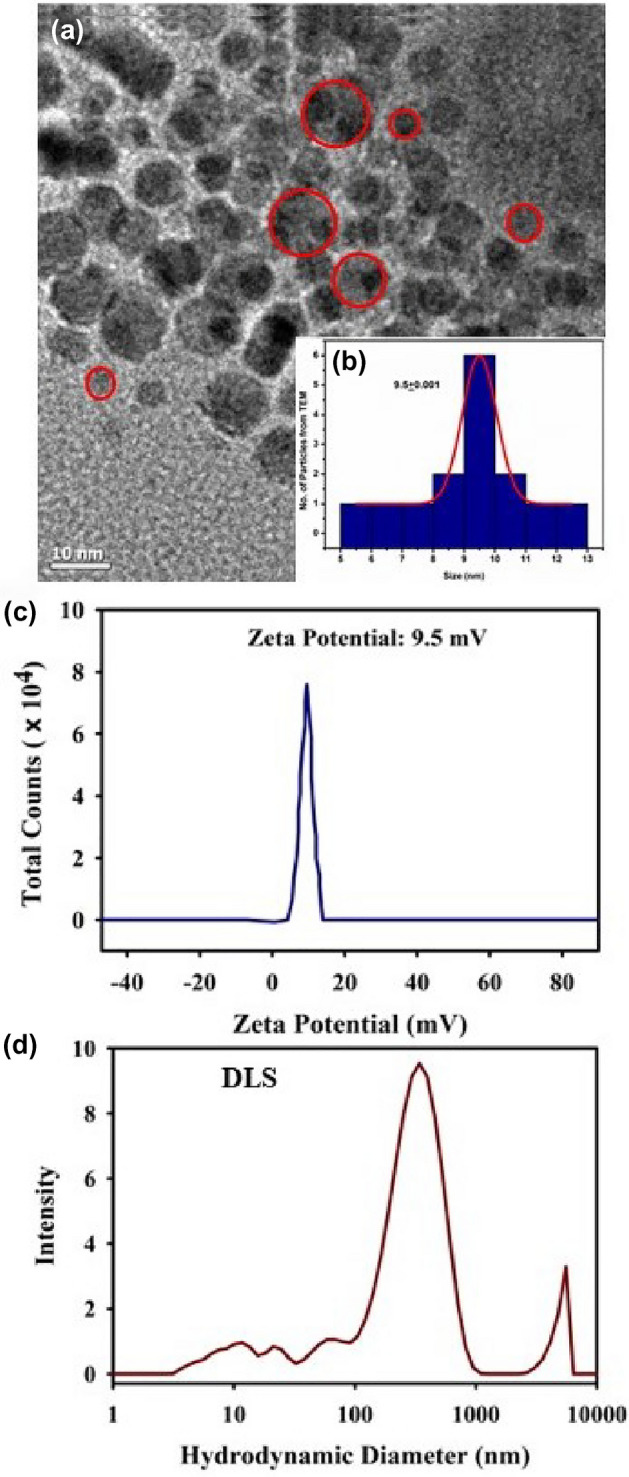
Characterization of Chitosan nanoparticles (**a**) TEM image, (**b**) the size distribution collected from HRTEM, (**c**) zeta potential, and (**d**) DLS.

Ch-Mn_3_O_4_ NPs are nanohybrids, composed of an organic ligand chitosan and metal nanoparticles. It is prior evidenced that metals, metal oxides, complex composites have been used as electro-catalysts in recent biosensing and diagnostic researches. Metal oxides possess few advantages as easy synthesis, low cost, functional biocompatibility, chemical stability, and most importantly enhanced electron transfer kinetics which make it a potential electro-catalyst^44^. These are the motivations behind considering Mn_3_O_4_ as a core of the nanoparticle. Functionalization of Mn_3_O_4_ nanoparticle with chitosan molecules generates many novel properties^45,46^. The uncapped or bare Mn_3_O_4_ nanoparticle is insoluble in water^47–49^. The uncapped Mn_3_O_4_ nanoparticle can be dispersed in aqueous solution but precipitates out from the solution with time. The stability of the uncapped nanoparticle was monitored in the aqueous solution and the uncapped nanoparticles were found to precipitate out from solution (Fig. 2a)^48,50–52^. Capping of Mn_3_O_4_ with ligand (here, functionalization with chitosan) makes the insoluble Mn_3_O_4_ to be soluble in aqueous media. In an earlier study^49,53^ using HRTEM, it has been shown that, the size, shape and uniformity of the uncapped one is similar to the capped nanoparticle but the physico-chemical properties of these two species are very different. The absorption spectrum of bare nanoparticle does not exhibit d-d band or any other distinguishable absorption peak (Data not shown). The uncapped Mn_3_O_4_ nanoparticle cannot generate ROS at room temperature but chitosan capped Mn_3_O_4_ nanoparticle effectively generates ROS without any external stimuli (Fig. 2b). This redox property of the NP is very important for the conversion of TMB i.e., for its nano-enzymatic action. The structural characterization of the aqueous solution of the synthesized Chitosan functionalized Mn_3_O_4_ nanoparticles have been conducted by performing TEM, zeta potential, DLS and FTIR, Raman Scattering study which are shown in the Fig. 3. The TEM image of Ch-Mn_3_O_4_ NPs presented in Fig. 3a depicts the shape as almost spherical. The higher-resolution TEM (HRTEM) indicates the crystalline nature of the nanoparticle. The inter-fringe distance between two planes of Mn_3_O_4_ is found to be 0.49 nm that represents the (101) plane of Mn_3_O_4_ spinel lattice (Fig. 3b). Figure 3c shows the size distribution of the nanoparticles to be uniform and ranges from 3.25 nm to 6.25 nm with an average diameter of 4.535 $\pm$ 0.71 nm (inset Fig. 3c). Zeta potential (Fig. 3d) reveals that the surface charge of the Ch-Mn_3_O_4_ NPs is 22.5 $\pm$ 9.34 mV (cationic). The diameter distribution of the Ch-Mn_3_O_4_ NPs is depicted in Fig. 3e. The achieved dominant particle size is 24.4 nm (hydrodynamic diameter). As mentioned previously, bare Mn_3_O_4_ without capping is not soluble in water, thus DLS and Zeta potential for Mn_3_O_4_ measurements without functionalization are not performed in our study. In order to cater the information about the attachment between the chitosan moiety and the Mn_3_O_4_ NPs, FTIR studies have been performed for the Mn_3_O_4_ NPs, Chitosan polymer, and Ch-Mn_3_O_4_ NPs respectively which are presented in Fig. 3f. Characteristic vibrations of Mn_3_O_4_ and Chitosan polymer are present and significantly perturbed in the synthesized Ch-Mn_3_O_4_ NPs. From Fig. 3f it can be concluded that the N–H stretching (3290 cm^−1^) and O–H stretching (3345 cm^−1^) of chitosan and vibration of O–H surface group of Mn_3_O_4_ have been shifted to 3021 cm^−1^ for Ch- Mn_3_O_4_ NPs^54^. Also, the C–H asymmetric stretching (2852 cm-1) of chitosan has been restricted in Ch-Mn_3_O_4_ NPs^55,56^. The vibrational bands responsible for C=O stretching (1653 cm^−1^) of amide I, N–H bending (1576 cm^−1^) of primary amine, CH2 bending (1423 cm^−1^), and C–N stretching (1320 cm^−1^) of Amide III of chitosan are present in the Ch-Mn_3_O_4_ NPs but are red-shifted in the EM spectrum^57^. C–O–C asymmetric bridge stretching (1155 cm^−1^), C–O stretching (1028 cm^−1^, 1072 cm^−1^), and C–H bending out of the plane of monosaccharide ring (891 cm^−1^) are present in the Ch-Mn_3_O_4_ NPs^58,59^. One of the characteristic absorbance bands comes for coupling modes between the Mn–O stretching mode of octahedral and tetrahedral sites of Mn_3_O_4_ nanoparticles is present in Ch-Mn_3_O_4_ NPs at 773 cm^−1^^60^. Therefore, FTIR results confirm the chitosan capping of Mn_3_O_4_ nanoparticles^61^. Figure 3g shows the Raman spectra of Chitosan and Ch-Mn_3_O_4_ NPs. Chitosan has characteristic Raman bands in the experimental region. Deformation bands of the aliphatic chains of chitosan gives the Raman band near 957 cm^−1^, amide III is responsible for the peak near 1445 cm^−1^ and peaks between 2800 and 3100 cm^−1^ are due to the C–H stretching in chitosan^62–64^. All these characteristic Raman bands of chitosan are prominent when it is capped around the Mn_3_O_4_ nanoparticle. Here, the surface enhanced Raman scattering (SERS) effect of chitosan reveals the characteristic peaks of chitosan while it is adsorbed on the Mn_3_O_4_ NPs^65^. These results provide the clear view on the surface modification of Mn_3_O_4_ NPs by the chitosan functionalization. Figure 4 represents the structural characterization of chitosan nanoparticle (as a control). Figure 4a shows the TEM of chitosan NPs which illustrates the amorphous characteristics of the chitosan polymer. Figure 4b,c provide the Zeta potential (9.5 mV) and DLS of the chitosan nanoparticles respectively. From Fig. 4c the multimodal size distribution of chitosan capping agent is clear compared to that of the chitosan capped Mn_3_O_4_ NPs (Fig. 3e) reveling essentially unimodal size distribution peaking at 24.4 nm.

### Enzymatic activity and sensing mechanism of the prepared Ch-Mn_3_O_4_ NPs

**Figure 5:**
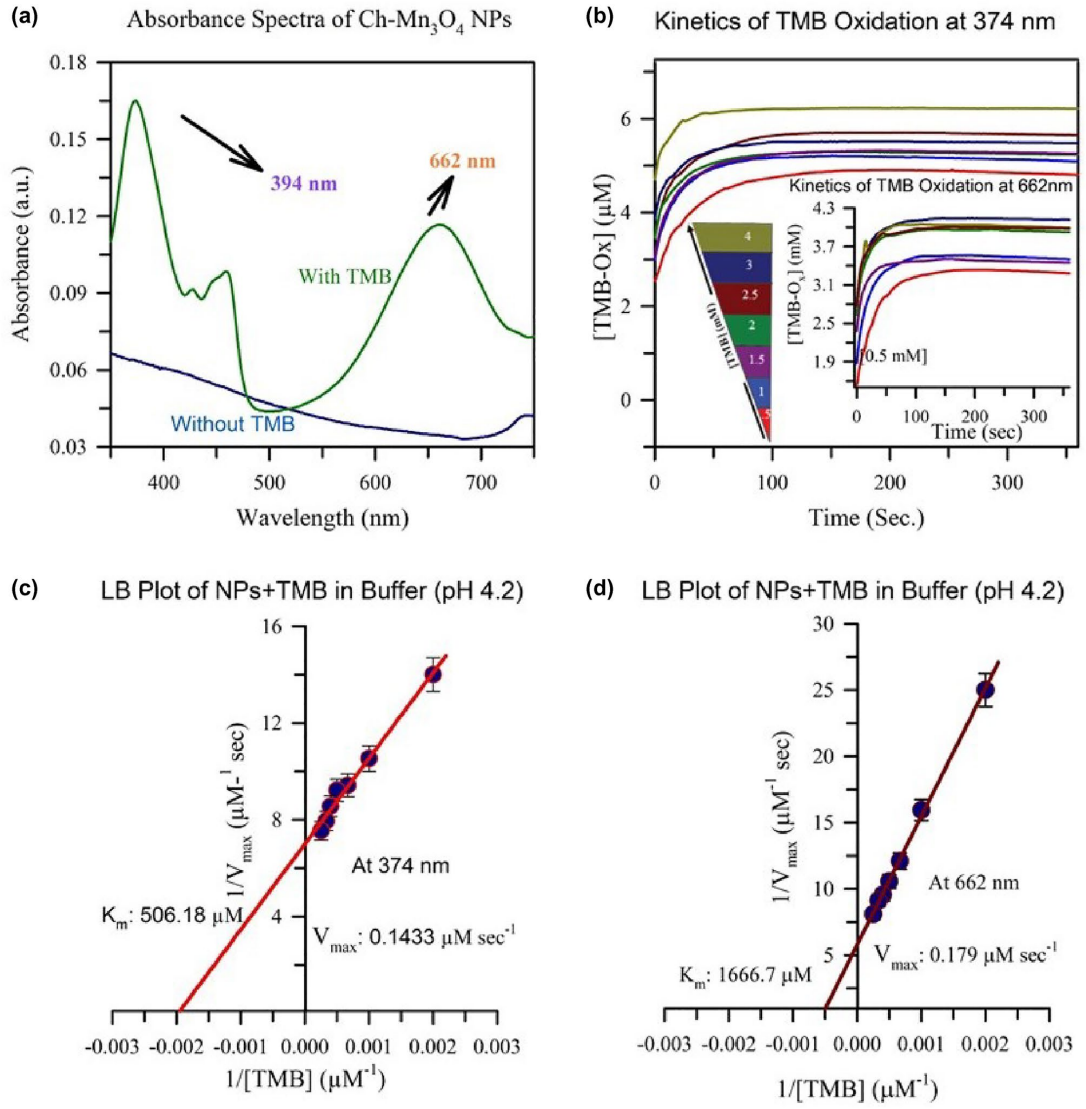
Absorbance spectra of the Ch-Mn_3_O_4_ in presence of acidic buffer (pH4.2) with and without TMB (substrate) is shown in figure (**a**). The reaction kinetics of the oxidation of TMB (0.5 mM-4 mM) by the nanoparticle have been shown at 374 nm and 662 nm (inset) in figure (**b**) and the corresponding Lineweaver Burk (LB) plots are depicted in figure (**c**) and (**d**).

**Figure 6:**
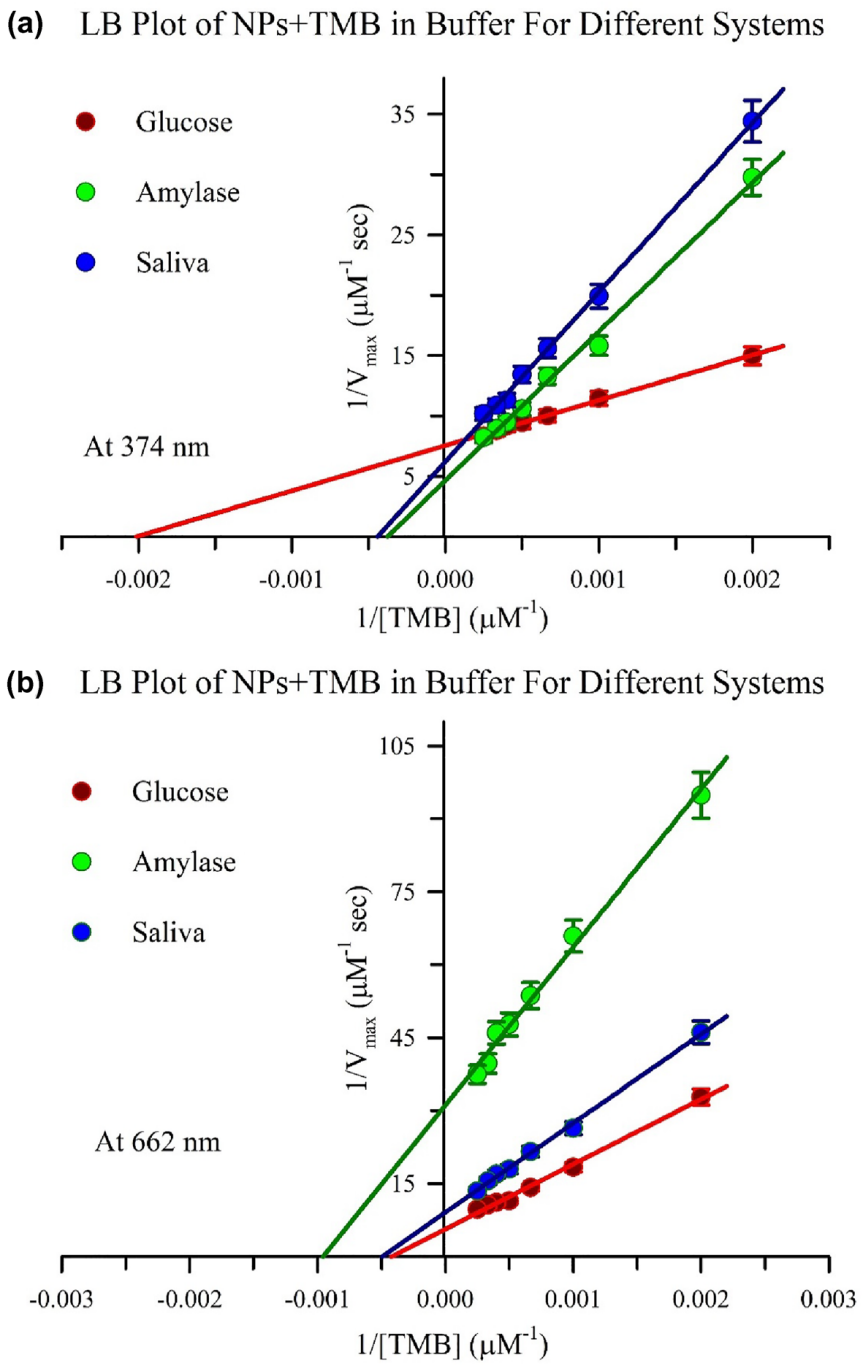
Lineweaver Burk (LB) plots for different analytic systems (glucose, amylase, and saliva) for two wavelengths, 374 nm, and 662 nm have been shown in figure (**a**), and (**b**) respectively.

Prepared Ch-Mn_3_O_4_ NPs are catalytic in nature in the acidic media (for experiment Sodium acetate buffer of pH 4.2 is used). It catalyzes oxidizing reaction to produce H_2_O_2_ from glucose and **·**OH from H_2_O_2_ along with oxidizing another substrate in the system, similarly as it happens for the GOD-POD reaction in glucose detection. GOD (glucose oxidase) produce gluconic acid and H_2_O_2_ from glucose and POD (peroxidase) oxidize 4-aminoantipyrine in presence of H_2_O_2_ and phenol to produce red colored quinoneimine dye. The developed nanozyme (Ch-Mn_3_O_4_ NPs) provides “enzyme-less” enzymatic detection technique for the detection of diabetic markers in saliva. To cater the enzymatic activity of Ch-Mn_3_O_4_ NPs 3,3′,5,5′-Tetramethylbenzidine (TMB) is being used as a colorimetric substrate. The NPs oxidize the colorimetric substrate, TMB in low pH and give rise of two peaks at 374 nm (double oxidation of TMB), and 662 nm (single oxidation of TMB)^66–68^ which is presented in Fig. 5a. The rate of oxidation with increasing concentration of TMB has been conducted for two absorbance peaks of oxidized TMB (TMB-O_x_). The kinetics of formation of TMB-O_x_ at wavelength 374 nm has been shown in the Fig. 5b, where TMB-O_x_ formation kinetics at 662 nm has been depicted in its inset. Lineweaver–Burk (LB) Plot for this two kinetics at two different absorbance peaks of TMB-O_x_ have been presented in Fig. 5c,d respectively. From L-B plot the reaction kinetic parameters (K_m_, V_max_, k_cat_) are determined. Enzyme kinetic parameters’ values are tabulated in Table 2. The formation of TMB-O_x_ by Ch-Mn_3_O_4_ NPs in presence of different analytes effects the enzymatic ability of the NPs, in other words increased concentration of TMB-O_x_ in the reaction medium alters the reaction parameters differently for different analytes. This variation of the catalytic activity of the Ch-Mn_3_O_4_ NPs makes it a nano-enzyme or simply a nanozyme and the difference in the reaction parameters are the building block of the sensing mechanism. In this study, Glucose, Amylase and saliva (in-vivo) act as the analytes for the detection of diabetes disease. It was observed that the velocity of the product formation and the binding affinity of the nanozyme with amylase and saliva are comparably same while the binding affinity of the nanozyme with glucose is higher at 374 nm. At 662 nm the rate of the reaction is highest for glucose whereas the binding affinity with the nanozyme is lowest. Although the binding affinity of amylase with Ch-Mn_3_O_4_ nanozyme at 662 nm is higher than saliva but the rate of the reaction is higher for saliva. The LB plot for 374 nm and 662 nm is depicted in Fig. 6a,b. The detailed reaction parameters for the three analytic system and for the two wavelengths are accumulated in Table 2. By looking into the Table 2 and the experimental results of Figs. 5 and 6, the inhibiting nature of the different analytes (glucose, amylase, and saliva) can be determined. This inhibiting nature of any of the analytes (substrates) mentioned above, indicating that the mechanism behind the enzymatic activity to be a Bi Bi reaction. In the end of the enzymatic reaction, we are getting oxidized TMB as the only generated product and the sequence of the presence of TMB or any of the analytes does not alter the end product. So, it could be the Random Sequential types of enzymatic reaction. The developed nanozyme (Ch-Mn_3_O_4_ NPs) is very selective to analytes. It is evidenced from the selectivity analysis that the nanozyme does not respond to other simple sugars such as galactose or fructose (data are not shown) It detects only glucose, and amylase in saliva.

**Table 2:** Reaction kinetic parameters for the catalytic reaction in enzyme activity assay.

System	Wavelength (nm)	K_m_ (µM)	V_max_ (µM s^−1^)	k_cat_ (s^−1^)
NP + TMB	374	505	0.142867	2495.6
	662	1.67E+3	0.1728	3036.9
NP + TMB + Glucose	374	495.05	0.132397	2326.8
	662	1.96E+3	0.1776	3121.3
NP + TMB + Amylase	374	2.65E+3	0.214856	3778.5
	662	1.05E+3	0.0321	564.14
Np + TMB + Saliva	374	2.37E+3	0.160981	2829.5
	662	2.97E+3	0.1101	1934.9

### Nanozyme functionalized microfluidic paper-based sensing mechanism

**Figure 7:**
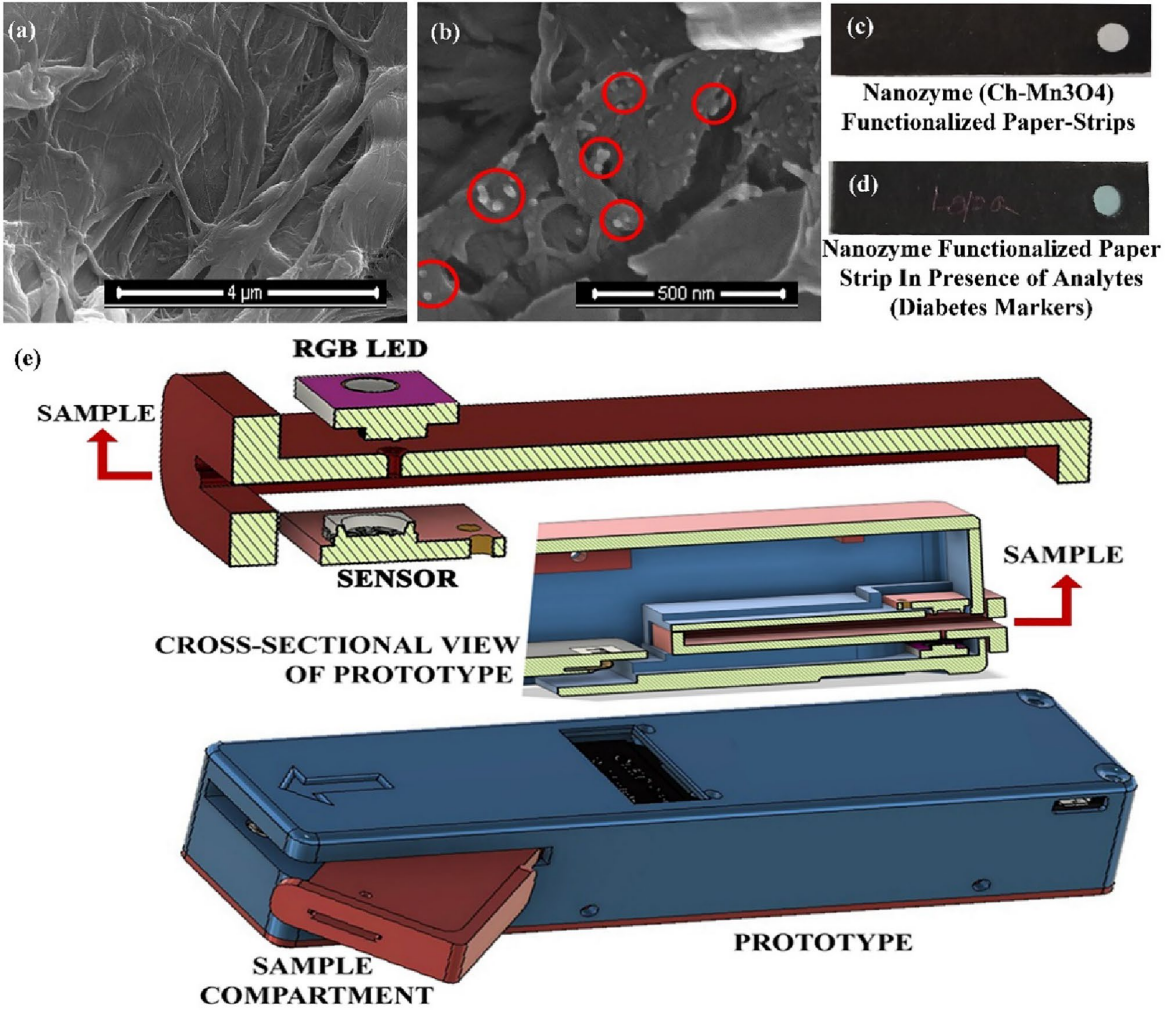
(**a**) Presents the SEM image of bare cellulose fibrous (Watman grad-4 filter paper) and (**b**) represents the Ch-Mn_3_O_4_ functionalized paper substrate. (**c**) shows the functionalized paper substrate after jacketing and (**d**) shows the nanozyme functionalized paper-strip in presence of analytes (glucose or amylase or saliva). (**e**) represents the 3-D schematic of the developed prototype for the detection of diabetic markers using the nanozyme functionalized paper-strips.

The sensing method of diabetes using the nanozyme can be easily transformed in the simple, easy and affordable paper-based microfluidics technology, as because the organo-metallic nanoparticle which is the nanozyme (Ch-Mn_3_O_4_) is cationic and the surface charge of the paper substrate which is made of cellulose fiber mesh is anionic^69,70^. Therefore, the adsorption of the Ch-Mn_3_O_4_ NPs with cellulose fiber is robust which in turn makes the paper-sensor stable^71^. Due to the opposite charge attraction a uniform layer of nanozyme forms on the cellulose filter paper which helps to produce a homogenized and uniform distribution of colorimetric information. This uniformly distributed information increases the signal quality during the detection process. Figure 7a,b show the SEM images of bare filter paper and Ch-Mn_3_O_4_ functionalized filter paper respectively. SEM shows the fibrous morphology of cellulose fibers in Fig. 7a, and in Fig. 7b the attached nanoparticles clearly can be observed. Whatman Grade-4 filter paper has been used as the paper-sensor substrate. Figure 7c,d show the packaged form of nanozyme functionalized paper-sensor without and with analyte and buffer (pH 4.2) respectively. For Fig. 7c the paper color is white as because it contains only the nanozymes on it but color of the paper sensor in Fig. 7d is blue as it contains acetate buffer along with nanoparticle and any of the diabetes markers which we have shown also previously that in presence of the low pH buffer the NPs behaves as nanozyme. The blue color comes from the oxidation of TMB (Combined effect of the absorbance at 374 nm and 662 nm) by the Ch-Mn_3_O_4_ NPs. This imparts that the catalytic activity of the nanozyme remains uninterrupted on the paper-based substrate. Therefore, Whatman Grade-4 filter paper is a potential non-reactive substrate material for our experimental conditions. The intensity of the blue color decreases with the increasing analyte concentration for a fixed nanozyme and acetate buffer concentrations. It has been observed, glucose or amylase behaves as inhibitors for the nanozyme. Our saliva contains glucose, and amylase which are the key targets molecules for a diabetic patient. Therefore, an increase in the amount of any of the components in saliva will decrease the intensity of the developed blue color on the paper-sensor. It is evidenced in prior literature that the diabetic patient has a larger amount of amylase in their saliva and the larger amount of amylase indicate directly the HbA1c parameter^72^. Higher the amylase, higher is the HbA1c value in the diabetic patients. So, using this nanozyme functionalized paper-sensor, the HbA1c and glucose amount can be determined from the saliva.

### Development of a prototype for colorimetric detection of Diabetes from human saliva

To gather the colorimetric information from the functionalized paper substrate at point-of-care, a prototype has been developed which is depicted in Fig. 7e. An RGB LED light source (CJMCU-123nWS2811) and an RGB color sensor (CJMCU-TEMT6000) has been arranged in I-geometry (the angle between them is 180°) to work in transmission mode. The sample chamber is in between the source and the sensor. Figure 7e also shows the cross-sectional view of the packaged prototype and depicts the 3-D schematic presentation of the prototype. Unprocessed data obtained from the color sensor was collected through a microcontroller arrangement (ESP-WROOM-32) for further processing. A small display screen is attached on the top of the prototype. The entire prototype functioning is controlled by indigenously developed software on the Arduino platform and attached with a 5 V rechargeable power supply. The dimension of the developed prototype is 176 mm × 38 mm × 35 mm. The designed model of the prototype discards unwanted light other than the sample information. The IOT enabled prototype increases the efficiency of data collection by enhancing the data storage and minimize the probability of catastrophe due to late diagnosis.

### Device calibration and validation

**Figure 8:**
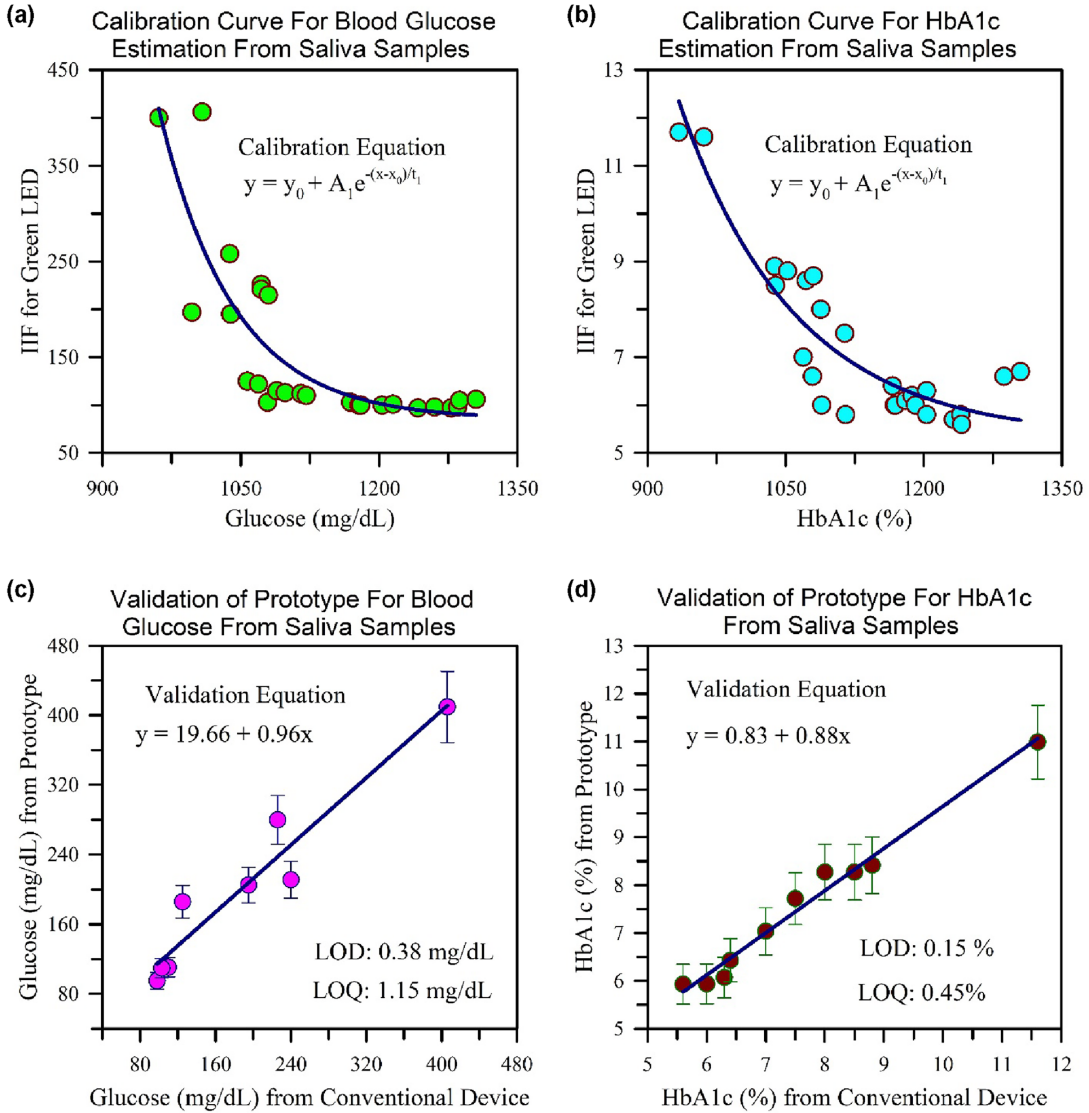
(**a**,**b**) represents the calibration curves of the prototype for blood glucose and HbA1c from the saliva samples of 26 subjects, and (**c**,**d**) are the corresponding validation curves.

Optimization of the substrate TMB and nanozyme concentrations have been performed to calibrate the prototype and also, the required working area (circular area of diameter 2.5 mm) and total volumetric capacity of liquid material (20 µL) on the nanozyme functionalized sensor have been optimized. In the case of colorimetric sensing of diabetes-related analytes (glucose, amylase) in human saliva, on functionalized paper, the used buffer is of pH 4.2. The nanozyme is an effective in acidic medium. The calibration experiment of the prototype was performed using the saliva samples of 26 subjects. The response (Instrumentation Index Function, IIF) of the prototype is collected for 26 subjects with known blood glucose and HbA1c values respectively. Nanozyme functionalized paper-sensor exposed with saliva and acetate buffer for 1.5 min before data collection. For each saliva sample, IIF for Red, Green, and Blue LED is noted and three sets of data have been collected. Figure 8a,b show the calibration graph of the prototype for blood glucose value (in mg/mL) and HbA1c (in %) value from the saliva samples. To calibrate the device the IIF for Green LED has been considered. The sensor in the developed prototype is saturated by the high intensity of the red light of the RGB LED owing to the less scattering property of the red light. The reverse phenomenon occurs in the case of blue light (high scattering property), where the low intensity received by the sensor results in erroneous data. However, for green wavelength, the information which is being collected are reliable. The duration of the one run (experiment) through the prototype is about 1 min. The calibration curve for the blood glucose and HbA1c detection, both obey the following equation,

$${\varvec{y}}={{\varvec{y}}}_{0}+{{\varvec{A}}}_{1}{{\varvec{e}}}^{-({\varvec{x}}-{{\varvec{x}}}_{0})/{{\varvec{t}}}_{1}}$$

Corresponding y_0_, A_1_, t_1_, and x_0_ for both calibrations have been tabulated in Table 3. Thereafter the calibration, the validation of the prototype using a drop of saliva sample on the nanozyme functionalized paper sensor has been performed to detect blood glucose and HbA1c values which are shown in Fig. 8. All the data are within 7% error range. We have estimated the limit of detection and limit of quantitation (LOD and LOQ) following well documented literature^73^. We considered LOD = (3.3 × σ)/slope and LOQ = (10 × σ)/slope. While the obtained σ and slope values from Fig. 8c for glucose are 0.11 mg/dL and 0.96 respectively, the corresponding values from Fig. 8d for HbA1c are found to be 0.04% and 0.88 respectively. The obtained LOD and LOQ for the sugar measurement in our developed prototype are 0.38 mg/dL and 1.15 mg/dL respectively. For the measurement of HbA1c through the amylase activity in saliva the obtained LOD and LOQ values are found to be 0.15% and 0.45% respectively. The accuracy of the device along with the sensitivity, specificity, positive predicted value (PPV) and negative predicted value (NPV) for both the estimation of glucose and HbA1c in 26 patients have been calculated. For the estimation of glucose, the sensitivity of the proposed instrument was found to be 100% with a specificity of 62.5%. The PPV and the NPV of the device for prediction of diabetes was found to be 62.5% and 100% respectively. This necessarily means that the number of false negatives predicted by the instrument is minimum and can accurately differentiate the diabetic population from the normal population. For estimation of HbA1c, the instrument has a sensitivity of 100% and a PPV of 68.4%. As the cut off for HbA1c was considered to be 5.7 based on the guidelines provided by the Centers for Disease Control and Prevention, the specificity and the NPV was found to be 53.8% and 100%, respectively. This necessarily means that the device has a zero NPV i.e., the chances of missing out on the disease population is minimum^74^. The development of a spectroscopy-based novel technique and establishing of the proof of the concept on limited number of the human subject is the main motive of the present work. Development of a functional prototype and its validation to a larger number of human subjects is out of the scope of the present work and motive of our future work which is progressing in our group.

**Table 3:** Fitting parameters of calibration and validation curves for the developed prototype device.

Fitting parameters	$y={y}_{0}+{A}_{1}{e}^{-(x-{x}_{0})/{t}_{1}}$ (Calibration curve)	
	Glucose	HbA1c
y_0_	85.29	5.32
x_0_	964.69	953.01
A_1_	309.77	6.04
t_1_	80.27	125.56

## Conclusions

The study aims to replace the conventional and painful blood test for the detection of diabetes. The enzymatic nature of the synthesized nano-enzyme is confirmed by Michaelis Menten kinetics on a chromogenic dye molecule TMB. We have confirmed that the salivary glucose and amylase have strong correlation with the blood glucose and glycated haemoglobin (HbA1c) respectively in the whole blood. We have also designed and fabricated a portable cost-effective prototype comprising the nano-enzyme on a covered cellulose film in the form of diagnostic strip for the detection of the diabetes markers from the saliva at the point of care setting. In a limited clinical trial, the prototype has been calibrated, evaluated, and found to be efficient to detect the diabetic markers without the requirement of blood test. To our understanding, our studies would find relevance in the management of blood sugar in the “diabetes epidemic” in the near future.

## Materials and methods

### Chemicals

All of the chemical reagents, such as manganese chloride tetrahydrate (MnCl_2_‚4H_2_O), ethanol, amylase, D-glucose, TMB, ethanol amine (EA), glacial acetic acid, chitosan and sodium acetate are of analytical grade and purchased from either from Sigma Aldrich or Merck. The chemicals have been used without further processing. The used water is from Millipore system. Whatman filter paper Grade 4 is from Sigma-Aldrich. Glucometer from Accu-Chek, China. HbA1c kit, Aina from Spark Diagnostics Pvt. Ltd, Gujrat, India.

### Synthesis of Ch-Mn_3_O_4_ nanoparticles

In this study a prior reported template-free sol–gel method have been used to synthesis Mn_3_O_4_ NPs at room temperature and pressure^53^. Synthesized Mn_3_O_4_ NPs is then functionalized with ligand chitosan. Chitosan solution with concentration of 5 mg/mL is being prepared in 1% acetic acid for the capping purpose. During the functionalization of Mn_3_O_4_ with chitosan the pH is adjusted to 6. The mixture of Mn_3_O_4_ and chitosan is kept for 24 h in cyclometer for stirring and then filtered using syringe filter to separate out the functionalized nanoparticle from the bulk solution. Prepared concentration of the Ch-Mn_3_O_4_ NPs is 0.06 nM. In recent times several works using chitosan and Mn_3_O_4_ have given their impact on research. For example, Mn_3_O_4_ Micro-octahedra had been used as cataluminescencing sensor for Acetone which is one of the diabetic markers^75^. Additionally, chitosan nanoparticles had been used as polymeric nano-carriers for the delivery of rifampicin^76^. On the other hand, chitosan- Mn_3_O_4_ had been used for remediation of several inflammatory diseases including IDB^77^. It has to be noted that no diagnostic devices have been employed using chitosan- Mn_3_O_4_ which is the main conceptual part of our present work.

### Characterization techniques

In this study, JASCO V-750 Spectrophotometer is used to collect the absorbance spectra. During the liquid state experiments, a quartz cuvette with an optical path length of 1 cm has been utilized. HRTEM images of the of the synthesized Ch-Mn_3_O_4_ NPs have been taken using FEI TecnaiTF-20 field emission HRTEM which operates at 200 kV to get the overview on the morphology and crystalline structure of the synthesized NPs. The NPs was drop on 300-mesh amorphous carbon-coated copper grids (Sigma, USA) and al-lowed to dry overnight at room temperature before the TEM image capturing. Fourier transform infrared spectroscopy (FTIR) of the Mn_3_O_4_ NPs, Chitosan, and Ch-Mn_3_O_4_ NPs have been performed in attenuated total reflectance (ATR) mode by Vertex 70 V instrument (Bruker, Germany) to look into the changes of the different vibrational bands of Mn_3_O_4_ NPs after capping with chitosan polymer was used to confirm the covalent attachment of the citrate molecules with the Mn_3_O_4_ NPs. Surface Enhancement Resonance Scattering (SERS) measurements has been performed in backscattering geometry using JobinYvon instrument which is fitted with a Peltier-cooled charge-coupled device detector. During SERS data collection a laser with a wavelength of 488 nm has been utilized as the excitation light source. Dynamic Light Scattering (DLS) and Zeta Potential of the Ch-Mn_3_O_4_ NPs is done by Zetasizer Nano S DLS instrument (Malvern, UK) (4mW, He–Ne laser, 632.8 nm, and a thermostatic sample chamber) to have the size distribution and surface charge of the synthesized Ch-Mn_3_O_4_ NPs respectively. SEM has been taken from QUANTA FEG 250, for the characterization of the cellulose paper substrate before and after nanozyme functionalization.

### Enzyme activity assay

To probe the enzymatic activity of the prepared nanozyme (Ch-Mn_3_O_4_), the Michaelis Menten kinetics was performed and Lineweaver–Burk plot was obtained. Here, 0.06 nM of nanozyme was used for different analytic (glucose, amylase, saliva) systems (behaves as second substrate in the system) and the used concentration range of the calorimetric dye TMB (substrate) for these analytical systems has been taken from 0.5 mM to 4 mM. The reaction kinetic parameters for the catalytic reaction such as K_m_, V_max_, k_cat_ have been determined.

### Ethical considerations

For the present work, all necessary ethical permissions were taken from the Institutional Medical Ethics Committee, Nil Ratan Sircar Medical College and Hospital (NRSMH), Kolkata (Ref. No.- No/NMC/443, Dated 26.01.2016). All studies involving human subjects were performed following the Declaration of Helsinki^78^ and guidelines provided by the Indian Council for Medical Research (ICMR), Govt. of India. All studies involving human subjects were performed following written informed consent obtained from the individual participants who agreed to participate in the study after understanding the details of the study and its consequences. All data and information about the subjects were anonymized and kept confidential, to be used only for this study.

### Sample collection

All the samples were taken from the human subjects following appropriate guidelines approved by the ethical committee, Nil Ratan Sircar Medical College and Hospital (NRSMH), Kolkata (Ref. No.- No/NMC/443, Dated 26.01.2016). The saliva samples were collected from a number of subjects across the country in the NRSMH, Kolkata. Before collecting the saliva samples after two hours of taking a meal, the subjects insisted to rinse their mouth cavities in order to avoid any extraneous substances. The subjects who are addicted to chewing several substances including tobacco were excluded during the sample collection. Therefore, the developed detection technique is very selective of the sugar or amylase content in saliva.

### Design and fabrication of the microfluidic paper-based sensor strips

Whatman Grade-4 filter paper is used as the paper substrate, firstly, because it is made of 98% cellulose fiber which decrease the contamination possibility, secondly, the homogeneous and fast flow rate provides a uniform layer on the paper substrate^79,80^. Here, 0.7 cm × 4 cm filter paper is covered in such a way so that it appears as a diagnostic sensor strip with a circular working area of 5 mm diameter. After jacketing, the filter paper is functionalized by drop casting the nanozyme (0.03 nM) and TMB (2 mM) onto the working area and dried to produce the final form of the sensor strip. For experiment with paper-strips, the analyte and acidic buffer (acetate buffer, pH 4.2) is being added one by one at a time).

### Procedure for the detection of glucose and HbA1c from saliva using prototype

For the detection of glucose and HbA1c in saliva which are the markers of diabetes, the prototype has been calibrated with human saliva. Prior calibration of the prototype the values of the two diabetes marks (blood glucose and HbA1c) have been obtained from ACCU-CHEK glucometer machine and Aina, HbA1c machine from the whole blood respectively. During calibration and validation 10µL of the saliva and 10 µL of acetate buffer are drop casted on the working region of the paper-strip and after drop casting the sample and buffer, the paper-strips are kept for 2 min before absorbance data collection.

## Data Availability

The datasets used during the current study are available from the corresponding author on reasonable request.
